# Supplementary material for: Quality of Life for Polish Women with Ovarian Cancer during First-Line Chemotherapy
Source: Healthcare (Basel). 2023 Sep 21;11(18):2596. doi: 10.3390/healthcare11182596 (PMC10530890; doi:10.3390/healthcare11182596)
Supplement: Supplementary file 1 [file healthcare-11-02596-s001.zip › healthcare-2587049-supplementary.pdf]

Supplementary Material

1.1. Perception of one's own body domain.

Table S1 presents the results of a multivariate analysis for the scale QLQ-OV28 in perception of one's own body domain.

The multivariate linear regression model showed that:

- age 50-60 reduces the perception of one's own body domain score by an average of 24,627 points in relation to age up to 50 years.

- age 61 and over reduces the perception of one's own body domain score by an average of 28,484 points in relation to age up to 50 years.

- FIGO stage III increases the result on the perception of one's own body domain by an average of 12.96 points in relation to stage II of FIGO scale.

Table S1. Women's quality of life according to the QLQ-OV28 in perception of one's own body domain at I stage

Variable		Regression coefficient	**95%CI**		**p**
Age	≤ 50	ref.			
	51-60	-24.627	-42.016	-7.237	0.007 *
	≥ 61	-28.484	-46.832	-10.136	0.003 *
Marital status	Single	ref.			
	Married	-1.447	-16.219	13.325	0.848
	Widow	-1.447	-17.443	14.55	0.86
Education	Elementary	ref.			
	Vocational	-1.88	-16.794	13.034	0.805
	Secondary	-0.767	-15.726	14.192	0.92
	Higher	-13.543	-31.851	4.766	0.151
Professional activity	Professionally active	ref.			
	Pension	-1.095	-18.023	15.834	0.899
Comorbidities	No	ref.			
	Yes	-2.023	-11.379	7.334	0.673
FIGO stage	II	ref.			
	III	12.96	2.135	23.785	0.021 *
	IV	6.119	-11.24	23.477	0.491

p - multiple linear regression*; statistically significant ($p<0.05$)

The R^2 coefficient for this model was 22.84%, which means that 22.84% of the variability of the perception of one's own body domain result was explained by the variables included in the model. (Table S1).

1.2.Sexuality domain

Table S2 presents the results of a multivariate analysis for the scale QLQ-OV28 in sexuality domain.

The multivariate linear regression model showed that: age 50-60 reduces the sexuality domain score by an average of 8.027 points in relation to age up to 50 years; age 61 and over reduces the sexuality domain score by an average of -9.657 points concerning age up to 50 years.

Table S2. Women's quality of life according to the QLQ-OV28 in sexuality domain at I stage

**Variable**		Regression coefficient	**95%CI**		**p**
Age	≤ 50	ref.			
	51-60	-8.027	-15.116	-0.939	0.029 *
	≥ 61	-9.657	-17.136	-2.178	0.013 *
Marital status	Single	ref.			
	Married	4.249	-1.773	10.27	0.17
	Widow	-1.448	-7.969	5.073	0.665
Education	Elementary	ref.			
	Vocational	-0.618	-6.697	5.462	0.843
	Secondary	-3.417	-9.515	2.681	0.275
	Higher	-1.335	-8.798	6.129	0.727
Professional activity	Professionally active	ref.			
	Pension	-5.25	-12.151	1.651	0.14
Comorbidities	No	ref.			
	Yes	-1.661	-5.475	2.153	0.396
FIGO stage	II	ref.			
	III	-3.87	-8.283	0.543	0.089
	IV	-3.048	-10.124	4.028	0.401

p - multiple linear regression;* statistically significant ($p<0.05$)

The R^2 coefficient for this model was 40.34%, which means that 40.34% of the variability of the sexuality domain result was explained by the variables included in the model. (Table S2).

1.3. Approach to disease/treatment domain

Table S3 presents the results of a multivariate analysis for the scale QLQ-OV28 in approach to disease/treatment domain.

The multivariate linear regression model showed that higher education reduces the result on the approach to disease/treatment domain by an average of 20.312 points compared to primary education.

Table S3. Women's quality of life according to the QLQ-OV28 in approach to disease/treatment domain at I stage

**Variable**		Regression coefficient	**95%CI**		**p**
Age	≤ 50	ref.			
	51-60	-13.417	-31.618	4.785	0.152
	≥ 61	6.713	-12.491	25.918	0.495
Marital status	Single	ref.			
	Married	0.82	-14.642	16.281	0.917
	Widow	4.874	-11.87	21.618	0.57
Education	Elementary	ref.			
	Vocational	-7.815	-23.425	7.796	0.329
	Secondary	-5.699	-21.356	9.959	0.478
	Higher	-20.312	-39.476	-1.148	0.041 *
Professional activity	Professionally active	ref.			
	Pension	-6.544	-24.263	11.175	0.471
Comorbidities	No	ref.			
	Yes	-3.89	-13.684	5.903	0.438
FIGO stage	II	ref.			
	III	10.656	-0.674	21.987	0.069
	IV	16.122	-2.047	34.291	0.086

p - multiple linear regression;* statistically significant ($p<0.05$)

The R^2 coefficient for this model was 19.87%, which means that 19.87% of the variability of the approach to disease/treatment domain result was explained by the variables included in the model. (Table S3).

1.4. Gastrointestinal symptoms domain

Table S4 presents the results of a multivariate analysis for the scale QLQ-OV28 in gastrointestinal symptoms domain.

The multivariate linear regression model showed that higher education reduces the result on the gastrointestinal symptoms domain by an average of 18.507 points compared to primary education.

Table S4.Women's quality of life according to the QLQ-OV28 in gastrointestinal symptoms domain at I stage

Variable		Regression coefficient	95%CI		p
Age	≤ 50	ref.			
	51-60	-6.861	-22.81	9.088	0.401
	≥ 61	3.054	-13.774	19.882	0.723
Marital status	Single	ref.			
	Married	-5.219	-18.767	8.329	0.452
	Widow	-4.163	-18.835	10.509	0.58
Education	Elementary	ref.			
	Vocational	-10.322	-24	3.357	0.143
	Secondary	-9.291	-23.01	4.429	0.188
	Higher	-18.507	-35.299	-1.715	0.033 *
Professional activity	Professionally active	ref.			
	Pension	4.378	-11.148	19.905	0.582
Comorbidities	No	ref.			
	Yes	1.5	-7.081	10.082	0.733
FIGO stage	II	ref.			
	III	5.594	-4.334	15.522	0.272
	IV	7.945	-7.976	23.865	0.331

p - multiple linear regression;* statistically significant (p<0.05)

The R^2 coefficient for this model was 20.40%, which means that 20.40% of the variability of the gastrointestinal symptoms domain result was explained by the variables included in the model. (Table S4).

1.5. Peripheral neuropathy domain

Table S5 presents the results of a multivariate analysis for the scale QLQ-OV28 in peripheral neuropathy domain.

The multivariate linear regression model showed that:
- secondary education reduces the result on the peripheral neuropathy domain by an average of 16.777 points concerning primary education.

Table S5. Women's quality of life according to the QLQ-OV28 in peripheral neuropathy domain at I stage

Variable		Regression coefficient	95%CI		p
Age	≤ 50	ref.			
	51-60	-2.808	-20.257	14.641	0.753
	≥ 61	-3.785	-22.196	14.626	0.688

Variable		Regression coefficient	95%CI		p
Marital status	Single	ref.			
	Married	-6.256	-21.078	8.566	0.41
	Widow	6.139	-9.913	22.191	0.456
Education	Elementary	ref.			
	Vocational	-12.93	-27.896	2.035	0.094
	Secondary	-16.777	-31.787	-1.766	0.031 *
	Higher	-16.965	-35.337	1.407	0.074
Professional activity	Professionally active	ref.			
	Pension	5.782	-11.205	22.769	0.506
Comorbidities	No	ref.			
	Yes	3.822	-5.566	13.211	0.427
FIGO stage	II	ref.			
	III	-3.585	-14.447	7.277	0.519
	IV	-9.426	-26.844	7.992	0.292

p - multiple linear regression;* statistically significant ($p<0.05$)

The R^2 coefficient for this model was 19.47%, which means that 19.47% of the variability of the peripheral neuropathy domain result was explained by the variables included in the model. (Table S5).

1.6 Hormonal /menopausal symptoms domain

Table S6 presents the results of a multivariate analysis for the scale QLQ-OV28 in hormonal /menopausal symptoms domain. The multivariate linear regression model showed that:

- age 50-60 reduces the score on the hormonal /menopausal symptoms domain
by an average of 17.118 points in relation to age up to 50 years;
- age 61 and over reduces the score on the hormonal /menopausal symptoms domain
by an average of 24.799 points in relation to age up to 50 years;
- widowhood reduces the score on the hormonal /menopausal symptoms domain
by an average of 13.8 points concerning virginity.

Table S6. Women's quality of life according to the QLQ-OV28 in hormonal /menopausal symptoms domain at I stage

Variable		Regression coefficient	95%CI	p
Age	≤ 50	ref.		

Variable		Regression coefficient	95%CI		p
	51-60	-17.118	-30.239	-3.996	0.012 *
	≥ 61	-24.799	-38.644	-10.954	0.001 *
Marital status	Single	ref.			
	Married	-3.481	-14.627	7.666	0.542
	Widow	-13.8	-25.871	-1.729	0.028 *
Education	Elementary	ref.			
	Vocational	0.719	-10.535	11.973	0.901
	Secondary	-2.402	-13.69	8.886	0.678
	Higher	-7.534	-21.35	6.281	0.288
Professional activity	Professionally active	ref.			
	Pension	-3.094	-15.868	9.68	0.636
Comorbidities	No	ref.			
	Yes	-0.494	-7.555	6.566	0.891
FIGO stage	II	ref.			
	III	8.032	-0.137	16.2	0.057
	IV	0.814	-12.284	13.912	0.903

p - multiple linear regression;* statistically significant ($p<0.05$)

The R^2 coefficient for this model was 39.25%, which means that 39.25% of the variability of the hormonal /menopausal symptoms domain result was explained by the variables included in the model. (Table S6).

1.7. Other side effects of chemotherapy domain

Table S7 presents the results of a multivariate analysis for the scale QLQ-OV28 in hormonal /menopausal symptoms domain. The multivariate linear regression model showed that higher education reduces the score on the other side effects of chemotherapy domain by an average of 17.223 points about primary education.

Table S7. Women's quality of life according to the QLQ-OV28 in other side effects of chemotherapy domain at I stage

Variable		Regression coefficient	95%CI		p
Age	≤ 50	ref.			
	51-60	-1.276	-14.957	12.406	0.855
	≥ 61	6.668	-7.768	21.103	0.368
Marital status	Single	ref.			
	Married	4.088	-7.534	15.71	0.492

Variable		Regression coefficient	95%CI		p
	Widow	10.602	-1.984	23.188	0.102
Education	Elementary	ref.			
	Vocational	-8.582	-20.316	3.152	0.155
	Secondary	-9.222	-20.991	2.547	0.128
	Higher	-17.223	-31.627	-2.818	0.021 *
Professional activity	Professionally active	ref.			
	Pension	2.11	-11.209	15.429	0.757
Comorbidities	No	ref.			
	Yes	2.617	-4.744	9.978	0.488
FIGO stage	II	ref.			
	III	-0.304	-8.82	8.213	0.944
	IV	5.965	-7.692	19.622	0.394

p - multiple linear regression;* statistically significant ($p<0.05$)

The R^2 coefficient for this model was 26.59%, which means that 26.59% of the variability of the other side effects of chemotherapy domain result was explained by the variables included in the model.(Table S7).

1.8. Hair loss domain

Table S8 presents the results of a multivariate analysis for the scale QLQ-OV28 in hair loss domain. The multivariate linear regression model showed that:

- age of 50-60 years reduces the result on the hair loss domain by an average of 17.059 points compared to the age of up to 50 years;

- age 61 and over reduces the score on the hair loss domain by an average of 18.103 points compared to age up to 50;

- secondary education reduces the result on the hair loss domain

by an average of 19.65 points (because the regression parameter is -19.65) compared to primary education;

- higher education reduces the result on the hair loss domain by an average of 20.362 points compared to primary education.

Table S8. Women's quality of life according to the QLQ-OV28 in hair loss domain at I stage

**Variable**		Regression coefficient	**95%CI**		**p**
Age	≤ 50	ref.			
	51-60	-17.059	-33.19	-0.929	0.041 *
	≥ 61	-18.103	-35.123	-1.083	0.04 *

Variable		Regression coefficient	95%CI		p
Marital status	Single	ref.			
	Married	2.686	-11.016	16.389	0.702
	Widow	-0.369	-15.208	14.47	0.961
Education	Elementary	ref.			
	Vocational	-9.97	-23.805	3.865	0.161
	Secondary	-19.65	-33.526	-5.774	0.007 *
	Higher	-20.362	-37.346	-3.379	0.021 *
Professional activity	Professionally active	ref.			
	Pension	6.204	-9.499	21.908	0.441
Comorbidities	No	ref.			
	Yes	0.121	-8.558	8.8	0.978
FIGO stage	II	ref.			
	III	-2.064	-12.106	7.977	0.688
	IV	-2.634	-18.736	13.468	0.749

p - multiple linear regression;* statistically significant ($p<0.05$)

The R^2 coefficient for this model was 16.63%, which means that 16.63% of the variability of the hair loss domain result was explained by the variables included in the model. (Table S8).
